# Biological networks and complexity in early-onset motor neuron diseases

**DOI:** 10.3389/fneur.2022.1035406

**Published:** 2022-10-21

**Authors:** Matthew E. R. Butchbach, Rod C. Scott

**Affiliations:** ^1^Division of Neurology, Nemours Children's Hospital Delaware, Wilmington, DE, United States; ^2^Department of Pediatrics, Thomas Jefferson University, Philadelphia, PA, United States; ^3^Department of Biological Sciences, University of Delaware, Newark, DE, United States; ^4^Department of Psychological and Brain Sciences, University of Delaware, Newark, DE, United States; ^5^Neurosciences Unit, Institute of Child Health, University College London, London, United Kingdom

**Keywords:** motor neuron disease, network biology, complexity, therapeutics development, spinal muscular atrophy, peripheral neuropathy, amyotrophic lateral sclerosis

## Abstract

Motor neuron diseases (MNDs) are neuromuscular disorders where the spinal motor neurons–either the cell bodies themselves or their axons–are the primary cells affected. To date, there are 120 different genes that are lost or mutated in pediatric-onset MNDs. Most of these childhood-onset disorders, aside from spinal muscular atrophy (SMA), lack viable therapeutic options. Previous research on MNDs has focused on understanding the pathobiology of a single, specific gene mutation and targeting therapies to that pathobiology. This reductionist approach has yielded therapeutic options for a specific disorder, in this case SMA. Unfortunately, therapies specific for SMA have not been effective against other pediatric-onset MNDs. Pursuing the same approach for the other defined MNDs would require development of at least 120 independent treatments raising feasibility issues. We propose an alternative to this this type of reductionist approach by conceptualizing MNDs in a complex adaptive systems framework that will allow identification of common molecular and cellular pathways which form biological networks that are adversely affected in early-onset MNDs and thus MNDs with similar phenotypes despite diverse genotypes. This systems biology approach highlights the complexity and self-organization of the motor system as well as the ways in which it can be affected by these genetic disorders. Using this integrated approach to understand early-onset MNDs, we would be better poised to expand the therapeutic repertoire for multiple MNDs.

## Introduction

Neuromuscular diseases (NMDs) result in chronic, severe disability as well as early mortality and a high burden of disease. They also display clinical and genetic heterogeneity; in fact, there are currently 1,079 distinct NMDs and 608 known genes associated with these disorders (1). While each NMD is rare, they collectively have a prevalence of about 1 in 700. Motor neuron diseases (MNDs) are a group of NMDs where the spinal motor neurons–either the cell bodies themselves or their axons–are the primary cells affected (2). Those MNDs with early onset include spinal muscular atrophies (SMAs), juvenile forms of amyotrophic lateral sclerosis (ALS), distal hereditary motor neuronopathies (dHMNs) and axonal–or type 2–Charcot-Marie-Tooth (CMT) disease. There are approximately 120 genes to date that are lost or mutated in pediatric-onset MNDs. Most of these childhood-onset disorders, aside from proximal SMA, lack viable therapeutic options.

The majority of research on MNDs has focused on a single disorder, like SMA, and to develop a single-target therapeutic strategy specific for that disorder. This reductionist approach has been applied to understanding disease pathogenesis and therapeutics development as well as for care (3). This approach to SMA has been successful in that there are currently 3 therapies–nusinersen (4, 5), risdiplam (6) and onasemnogene abeparvovec (7)–that are approved for clinical use which target *SMN* expression, either by *SMN1* gene replacement or modulating the alternative splicing of the orthologous *SMN2* gene. While these options are likely to have significant positive impacts on the quality of life of many patients with SMA, not all SMA patients respond positively to these clinically approved therapies and the length of therapeutic benefit in these patients is not known (8). For maximal therapeutic benefits these agents need to be administered at the early stages of disease progression. These approved therapies lessen the severity of disease in SMA but, unfortunately, do not completely cure children with this disorder raising the issue of whether combination therapies could improve outcomes further. Importantly, therapies specific for SMA have not been effective against other pediatric-onset MNDs thereby limiting their benefits for early-onset MNDs in general. These issues suggest that there needs to be a broadening of concepts related to pathophysiology of MNDs with a view that this will lead to alternative research approaches and ultimately the development of novel therapies.

While each disorder is caused by a loss of or mutation in a specific gene, there are commonalities with respect to mechanisms of disease within the various early-onset MNDs as well as within other neurodegenerative diseases (9). Motor units are functionally linked entities composed of spinal motor neurons that innervate skeletal muscles as well as regulatory interneurons projecting from these target muscles. The question we pose in this article is whether motor units can be conceptualized as complex adaptive systems and whether there are pathophysiologic and therapeutic advantages to a complex adaptive system conceptualization.

## Motor neuron diseases and biological networks

The commonalities amongst the genetically distinct early-onset MNDs can be understood holistically using a network biology, or network medicine, approach. Network biology examines the interactions and interdependencies between biomolecular components within a system, as opposed to the components themselves (10). Examination of these networks within motor neurons provides important insights into their function as well the consequences of a disease-associated mutation within one of the components. This characterization can be completed at different levels including gene regulatory networks, protein-protein interaction networks as well as metabolite analysis (11, 12).

Interrogating the changes in gene regulatory networks has provided important insights into how motor neurons are affected in early-onset MNDs. A genetic screen of modifiers of *Smn* dependent lethality in *Drosophila melanogaster* identified pathways involved in alternative splicing, neuronal differentiation, axonal guidance and neuromuscular junction maturation as modifier of the SMA-like phenotype (13). Using a genetic screen on a fruit fly model of ALS8–caused by loss of functional *VAMP-associated protein B* (*VAPB*), genes associated with vesicular trafficking, endosome trafficking and cell proliferation were identified as modifiers of disease phenotype (14). Pathways involved in cell proliferation and neuronal development were differentially affected in SMA mouse motor neurons derived from embryonic stem cells (15). Network analysis of presymptomatic SMA mouse spinal cord transcriptomes showed impairment of neurotrophin signaling pathways (16). Comparing the transcriptomes of motor neurons vulnerable to neurodegeneration against those which are resistant to neurodegeneration in wild-type as well as SMA mice revealed enrichment in pathways associated with ribosome biosynthesis, translation, the extracellular matrix and cell adhesion (17, 18). Kline et al. (18) compared their list of transcripts that were differentially vulnerable to neurodegeneration with independent screens for motor neuron vulnerability transcripts (19–21) and identified 595 transcripts altered in the same direction between at least two of the modifier screens. Network analysis showed enrichment of pathways involved in neuronal function and synaptogenesis. A comparison of the transcriptomes of SMA spinal motor neurons that are vulnerable to neurodegeneration against those motor neurons which are resistant to degeneration (ocular motor neurons) revealed networks associated with neurotransmitter release, neuronal survival, oxidative stress and apoptosis as modifiers of neurodegeneration associated with the loss of functional SMN (22).

Weighted correlation network analysis (WGCNA) is an approach to identify disease-related networks and hubs from different datasets (23). When comparing the transcriptomes from SMA patients with varying degrees of phenotypic severities, WGCNA identified transcripts associated with tumor necrosis factor α (TNFα)-mediated regulation of neural, cardiac and bone development (24). These target transcripts showed similar disease severity-dependent differential expression in mouse models for SMA. Large-scale WGCNA of blood transcriptomes from ALS patients revealed enrichment of pathways involved in neurodegeneration and inflammation (25). The complement activator *dehydrogenase/reductase (SDR family) 4* (*DHRS4*) was identified as a hub gene for ALS1 by comparing the transcriptomes of *SOD1 (G93A)* mouse spinal cords using WGCNA (26). This analysis suggests the role of the complement cascade in motor neuron degeneration in ALS.

WGCNA can also be used to distinguish disease-specific network regulation between allelic disorders. For examples ALS4 and ataxia with oculomotor ataxia type 2 (AOA2) are clinical distinct early-onset neurodegenerative diseases disorders but are caused by mutations in *senataxin* (S*ETX*) (27, 28). WGCNA of fibroblast transcriptomes with ALS4-specific *SETX* mutation against those with an AOA2-specific *SETX* mutation identified networks which are unique to either disorder (29). Specifically, networks associated with the regulation of RNA metabolism were overrepresented in ALS4 transcriptomes. Comparison of spinal cord and cerebellum transcriptomes from two different mouse models for ALS4 [expressing either *SETX (R2136H)* or *SETX (L389S)* mutant transgenes] identified overrepresentation of pathways involved in synaptic signaling and nervous system development (30). Interestingly, these pathways were not enriched in a mouse model for AOA2.

In addition to gene regulatory network analysis, important insights into the pathogenesis of early-onset motor neuron diseases can be gained from the analysis of disease-associated protein-protein interaction (PPI) networks. When comparing the protein-protein interactomes between ALS and ALS linked with frontotemporal dementia (ALS-FTD), there were common hub proteins between these two types of motor neuron disease but the enrichment profiles around these hubs were distinct between ALS and ALS-FTD (31). This PPI network analysis identified ubiquitin-C as a common downstream target amongst these disorders. Jensen et al. (32) recently compiled a PPI network analysis for ALS and identified key modules associated with disease phenotype–oxidative stress, energy metabolism, proteosome dysfunction and mRNA processing defects. Some of these modules are commonly observed in other forms of neurodegeneration but other networks, like oxidative stress-induced protein misfolding, were found to be unique to ALS. A similar analysis of the ALS PPI networks showed similar modules and identified the cell cycle regulatory protein CDC5L as a stabilizer of these interactomes (33).

Biological networks have also been identified amongst disorders affecting motor neuron axons, such as type 2 CMTs and hereditary spastic paraplegias (HSPs). Network analysis of all CMT-related genes–including those linked to demyelinating peripheral neuropathy–identified pathways involved in myelination, axonal function, mitochondrial metabolism and autophagy as key pathways in CMT pathogenesis (34). Bis-Brewer et al. (35) identified common modules between the PPI networks of these axonopathies. These common modules include protein misfolding (endoplasmic reticulum stress) response, spliceosome assembly and function as well as mRNA processing. They also found that energy metabolism processes, mainly those related to glycolysis and gluconeogenesis, were unique to the type 2 CMTs. Comparison of the transcriptomes of different cellular models of type 2 CMT motor neurons {CMT2A2A [*MFN2(R94Q)]*, CMT2E [*NEFL(P8R)*], CMT2F [*HSPB1(G84R)* and *HSPB1(P182L)*] and CMT2L [*HSPB8(K141N)*]} revealed overrepresentation of mitochondrial respiratory chain and axon guidance networks (36). Interestingly, the differential overrepresentation of these networks is unique to motor neurons.

There is strong overlap and interconnectivity between the SMA and ALS PPI networks (37). One of the first common links between PPIs for SMA and ALS was described in 2017 where survival motor neuron 1 (SMN1) and the ALS-associated proteins FUS RNA binding protein (FUS), TAR DNA binding protein 43 (TDP43) and senataxin (SETX) were identified as shared components with the RNA metabolism network (38). Interactome analyses of the ALS-associated proteins FUS, EWS RNA binding protein 1 (EWSR1), TAT-box binding protein associated factor 15 (TAF15) and matrin-3 (MATR3) revealed a common association with the U1 snRNP assembly network as well as the RNA polymerase II network (39). The U1 small ribonucleoprotein (snRNP) assembly network is also linked to SMA. This group also demonstrated five proteins linked to SMA– SMN1, activating signal cointegrator 1 complex component 1 (ASCC1), exosome component 8 (EXOSC8) and heat shock protein B1 (HSPB1)–were integral components of this RNA polymerase II network (40). Comparative proteomics between SMA and ALS biosamples revealed alterations in energy homeostasis, protein metabolism and oxidative stress response that were present in both disorders (41). Kubinski and Claus (42) used network analysis to show that the ALS-linked protein Cu/Mn-dependent superoxide dismutase (SOD1) and profilin-1 (PFN1) serve as intermodular nodes connecting motor neuron disease pathogenesis and actin cytoskeleton dynamics.

Modifier genes can regulate disease phenotype without necessarily causing the early-onset MNDs. *SMN2* is an extremely well characterized modifier gene for SMA as increasing *SMN2* copy number lessens the disease severity [reviewed in (43)]. *Plastin-3* (*PLS3*)–one of the first *SMN2*-independent modifier genes identified for SMA–mRNA levels were found to be higher in females with milder SMA than those siblings with a more severe SMA clinical presentation (44–49). The modifier capability of *PLS3* for SMA was subsequently found to be incompletely penetrant within the SMA population as well as within animal models (47, 50–53). Other putative SMA modifier genes identified to date include *neurocalcin-D* (*NCALD*) (54), *Tolloid-like 2* (*TLL2*) (55) and *neuritin 1* (*NRN1*; cpg15) (48, 56). When comparing the clinical variability between CMT4K, *ganglioside-induced differentiation-associated protein 1* (*GDAP1*)-linked patients, *junctophilin-1* (*JPH1*) was identified as a modifier of disease phenotype by altering mitochondrial calcium channel activity (57). Loss of or mutation in *neuropilin-1* (*NRP1*), the voltage-gated sodium channel NaV1.6 (*SCN8A*) or *neuronal cell adhesion molecule* (*NRCAM*) are linked to disease severity in CMT4C–which is mediated by mutation in the *glycyl-tRNA synthetase* (*GARS*) gene (58, 59).

In addition to identifying common molecular pathways amongst early-onset MNDs, network analysis can provide important insights into therapeutic options. Using this systems network approach, ubiquitin-C was identified as a common therapeutic target between the various genetic forms of ALS and ALS-FTD (31). Gene delivery of *UCHL1*–a key enzyme controlling the amount of ubiquitin-C present in polyubiquitin chains on proteins–to upper motor neurons improves motor neuron phenotype in multiple models of ALS (60). Future studies will demonstrate the therapeutic potential of *UCHL1* modulation in multiple early-onset MNDs as well as identify *via* network analysis additional common targets for therapeutic benefit.

Sensory inputs onto motor neurons are also affected in early-onset MNDs. Mentis et al. (61) showed that SMA motor neurons lose their proprioceptive sensory inputs, referred to as deafferentation, during early stages of disease progression in the SMNΔ7 SMA mouse model. Deafferentation of motor neurons has been confirmed in a different mouse model for SMA (62). This loss of sensory input to motor neurons is a result of misregulation of *ubiquitin-like modifier-activating enzyme-1* (*UBA1*) and *GARS* (63). Both genes have been implicated in early-onset MNDs. It remains to be determined whether loss of sensory inputs to motor neurons is universal to early-onset MNDs. The biological networks implicated in deafferentation of motor neurons need to be more fully characterized to determine how they are affected in early-onset MNDs.

Biological networks affected by early-onset MNDs involve glial cells in addition to motor neuron [reviewed in (64)]. Reactive gliosis was observed in the spinal cords of patients with type I SMA (65, 66) as well as in pathological specimen from CMT2A2 patients (67). Reactive astrogliosis occurs during the end stage of disease progression in SMA mouse models and restoration of SMN in SMA astrocytes reduces the release of proinflammatory cytokines (68, 69). SMA reactive astrocytes show altered calcium homeostasis and hyperactivation of Notch signaling (70, 71). SMN-deficient reactive astrocytes alter the release of regulatory agents like MCP-1 (monocyte chemoattractant protein-1) and miR-146a which promotes motor neuron loss (72, 73). SMA astrocytes also show diminished ability to form and maintain neuromuscular synapses (74).

Microglia are activated within the spinal cord during early-onset MNDs. Microglial activation has been observed in mouse models for SMA (68, 75) as well as for CMT2K (76). SMA microglia acquire a reactive phenotype that enhances inflammatory responses in the spinal cord (77). Classical complement C1q pathways are activated in SMA to mark damaged motor neurons for microglial degradation (78). Microglial activation can be attenuated in SMA mice with the sigma-1 receptor agonist PRE-084 (79). PRE-084 can also attenuation deafferentation in SMA motor neurons. Future studies need to fully characterize how alterations in glial–both astroglial and microglial–reactivity can interact with and affect motor neuron biological networks.

## Lower motor units as complex adaptable systems

The previous section highlights the observation that a genetic mutation alters multiple networks in a way in which there is biological convergence with similarities in emergent phenotypes. We suggest that the convergences are critically important components of disease pathophysiology that deserve explicit study. A strong theoretical framework underlying these sorts of studies is that of complex adaptive systems.

**Figure 1:**
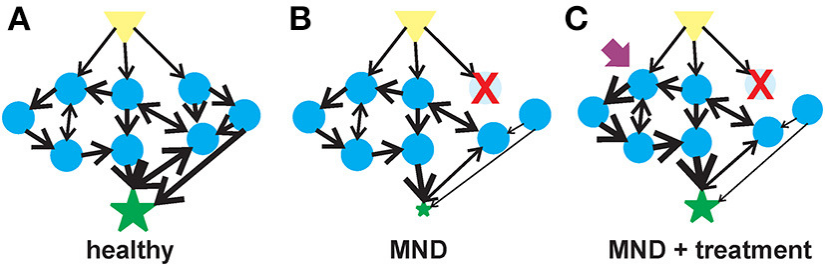
Flow of information in a complex system in motor neuron health, disease and therapeutic intervention. **(A)** In a healthy motor unit–lower motor neurons, interneurons and the muscle, activation of a signal (yellow triangle) leads to the stimulation of multiple agents (blue circles) that are interconnected into collection of aggregates known as meta-agents to form a hierarchical higher-order structure. The aggregates interact non-linearly to elicit a response (green star) whose behavior is quantitatively different from the individual agents. **(B)** Upon the loss of or alteration in an agent (in this case, an early-onset MND represented by the red X), the behavior of the aggregated meta-agents is altered and the flow of information is blunted. **(C)** A therapeutic agent (purple arrow) can activate one of the undamaged agents which would, in turn, activate the unaffected parts of the hierarchical aggregate and partially compensate for the diminished flow of information resulting from the altered agent.

A generalizable description of complex adaptive systems (Figure 1A) is that they are collections of relatively simple agents that have the property that they can aggregate, so that collections of agents can form meta-agents (and meta-meta-agents etc…) with hierarchical higher-order structure (80–82). These aggregates interact non-linearly, so that aggregate behavior of a collection of agents is qualitatively different from the behavior of the individual agents, e.g., the behavior of a collection of genes is qualitatively different to the behavior of a collection of neurons. The interactions among agents mediate flows of materials or information. Finally, the agents are typically diverse with distinct specialties that are optimized through adaptation to selective pressures in their environments. There are an enormous number of system types that meet these criteria e.g., economies, ecologies, traffic networks and brains (83, 84). Given the commonalities in underlying structure of these systems, there are generalizable strategies for understanding and manipulating such systems. The complex adaptive systems approach to biology is rather new, but the ideas resonate with many scientists and therefore studies designed within this framework are becoming more common. Our aim is to operationalize these general concepts to MNDs and explore how this would change current paradigms.

Motor units can easily be seen as complex adaptive systems. At the lowest hierarchical layer there is molecular diversity amongst spinal motor neurons (85). Networks of gene expression defined by transcriptomics non-linearly influence the emergence of spinal motor neurons that differ by temporal development and differentiation as well as by spatial organization within the body (86). Importantly, it is not the function of a single gene that determines motor neuron fate, but rather the interactome of genes and the non-linear hierarchical interactions of proteins, neurons and networks that define the emergent phenotypes we define a motor function. During development, spinal motor neurons differentiate into compact anatomic units known as motor neuron pools that connect to specific and unique muscle (87). The composition of these motor neuron pools is heterogeneous based on the types of myofibers they innervate. The diversity of motor neuron subtypes as well as their formation into pools suggests multiplicity and self-organization. Mathematical modeling of the spinal monosynaptic reflex unit as well as the regulatory interneurons demonstrate functional self-organization of the motor unit (88, 89). Thus, motor units can be characterized by non-linear interactions between multiple components operating on multiple temporal and spatial scales (90). High-density surface electromyographic (EMG) recordings of synergistic muscles in the thigh demonstrate both shared synaptic drive amongst the neural inputs within the synergistic group as well as synaptic control specific to a muscle, depending on the frequency of the neural drive (91). This synchrony between spinal motor neurons and muscles involved in movement starts manifesting itself during neonatal development (92). While there are common inputs controlling groups of motor neurons to produce movement, there is not complete concordance between motor neuron input and muscle anatomy (93). This incomplete concordance suggests non-linearity and multi-scaling properties of complex adaptive systems.

Disease states, like adult-onset MNDs and aging, arise from the breakdown of the structures underpinning physiologic complexity (Figure 1B) (94, 95). One manifestation of this breakdown of physiological complexity may be spontaneous or induced discharges of hyperexcitable motor units known as fasciculations (96). Fasciculations have been observed in adults with ALS as the disease progresses (97). There is a relationship between the frequency of these fasciculations and muscle type in ALS patients (98). Muscle fasciculations in ALS may be an early indicator of motor neuron dysfunction (99) although their usefulness as an indicator for motor unit dysfunction has been questioned (100, 101).

Are fasciculations observed in early-onset MNDs? Clinical characterization of SMA population found the presence of tremors in some patients (102–104). Muscle fasciculations have also been observed in men with spinal and bulbar muscular atrophy (SMAX1) with a possible relationship with disease severity, triplet repeat length with the *androgen receptor* (*AR*) gene and testosterone levels (105). As with cases of adult ALS, these fasciculations are not always present in patients with early-onset MNDs. Further detailed analyses of fasciculations in patients with early-onset MNDs will provide important insights into the network level drivers of disease progression.

## Multi-modal and multi-indication therapeutics for motor neuron diseases

Therapeutics options for early-onset MNDs are limited and have focused on targeted replacement of the specific MND-associated gene that is lost or mutated. While these targeted therapeutic replacement strategies have significantly improved the quality of life for patients with early-onset MNDs, treated individual still have a partial disease phenotype. As early-onset MNDs can be considered complex adaptive systems, therapeutic agents that target another component of the motor unit–aside from the one lost by genetic mutations–could provide significant therapeutic benefit (Figure 1C). There is mounting evidence demonstrating the efficacy of SMN-independent targets for SMA (106) and, by extension, other early-onset motor neuron diseases. These neuroprotective agents have multiple targets for their therapeutic benefits. The development of therapeutic strategies to manage epilepsy demonstrates the value of multi-target therapeutic strategies to treat neurological diseases (107). As examples of multi-target therapies for motor neuron diseases, we will describe two neuroprotectants that have shown strong *in vivo* efficacy in different types of motor neuron diseases: 4-phenylbutyrate (4PBA) and inhibitors of histone deacetylase 6 (HDAC6) (Table 1).

**Table 1:** Summary of 4-PBA and HDAC6 inhibitors demonstrating therapeutic benefit in model systems for early-onset motor neuron diseases.

**Compound**	**Disease model**	**Effect**	**Reference(s)**
4-PBA	SMNΔ7 mouse	Improvement in survival and phenotype	(108)
4-PBA	SMA patient-derived fibroblasts	Modest increase in FL-SMN expression; interindividual variability	(109–111)
4-PBA	SMA patients	Modest improvement in motor function	(112, 113)
4-PBA	*SOD1 (G93A)* mouse	Marked improvement in survival and motor function	(114)
4-PBA + riluzole	*SOD1 (G93A)* mouse	Additive ameliorative effects relative to 4PBA or riluzole alone	(115)
4-PBA + AEOL-10150	*SOD1 (G93A)* mouse	Additive ameliorative effects relative to 4PBA or AEOL10150 alone	(116)
4-PBA	Postsymptomatic ALS patients	Improved motor function	(117)
4-PBA + taurursodiol	Postsymptomatic ALS patients	Additive ameliorative effected related to 4PBA alone	(118)
Tubastatin A	*FUS (R521H)* and *FUS (P525L)* cultured motor neurons (ALS6)	Improves motor neurite outgrowth and function	(119, 120)
Tubastatin A	*TARDBP (A382T), TARDBP (G287S)*, and *TARDBP (N390S)* cultured motor neurons (ALS10)	Improves neurite outgrowth and mitochondrial transport along axons	(121)
Tubastatin A	*HSPB1 (S135F)* mouse (CMT2F)	Markedly improves motor neuron and muscle innervation	(122)
Tubastatin A	*GARS (R234KY)* and *Gars (C201R)* mice (CMT2D)	Improves motor function	(123)
Tubastatin A	*GARS1 (P724H)* cultured motor neurons (CMT2D)	Improves electrophysiologic misfiring and axonal transport defects	(124)
Tubastatin A	*Gars* knocked-down zebrafish embryos	Improve deficient neurite outgrowth and defective neuromuscular junction maturation	(125)
SW-100	*MFN2 (R94Q)* mouse (CMT2A1)	Improves motor phenotype	(126)
CKD-504	*GARS1 (P724H)* cultured motor neurons (CMT2D)	Improves electrophysiologic misfiring and axonal transport defects	(124)
CKD-504	*Gars* knocked-down zebrafish embryos	Improve deficient neurite outgrowth and defective neuromuscular junction maturation	(125)
ACY-738	*FUS (R521H)* and *FUS (P525L)* cultured motor neurons	Improves motor neurite outgrowth and function	(119)
ACY-738	*FUS* overexpressing mice	Improves motor function and diminishes neurodegeneration	(127)
ACY-738	*HSPB1 (S135M)* mice (CMT2F)	Improves survival and ameliorates the motor phenotype	(128, 129)
T3796106	Cultured *HSPB1 (S135M)* CMT motor neurons (CMT2F)	Improves motor neuron phenotype	(130)
T3793168	Cultured *HSPB1 (S135M)* CMT motor neurons (CMT2F)	Improves motor neuron phenotype	(130)

4PBA is one of the first multimodal therapeutics proposed from multiple motor neuron diseases. In SMA, early studies demonstrated that 4PBA increases *SMN2* expression in SMA patient-derived cells (109, 110), Administration of 4PBA to SMNΔ7 SMA mice resulted in a significant improvement in survival and motor function (108). As 4PBA was approved for treating humans, pilot clinical trials for 4PBA in SMA was completed with modest ameliorative effects in patients (112, 113). These studies did not detect any significant changes in *SMN2* expression in SMA patients treated with 4PBA. Additional testing of patient-derived SMA cell lines showed a high degree of interindividual variability in responsiveness to 4PBA (111). In the SMNΔ7 SMA mice, the protective effects of 4PBA—as well as of other butyrate prodrugs–were independent of *SMN2* induction (108). 4PBA significantly improves motor dysfunction and increases lifespan in the *SOD1 (G93A)* mouse model for ALS (114). In the same ALS mouse model, 4PBA displayed additive effects when co-administered with other neuroprotectants–such as riluzole and AEOL-10150–that have shown efficacy on the motor neurons of ALS mice (115, 116). In a clinical trial, 4PBA improved motor outcomes in a cohort of ALS patients who received the therapeutic after disease onset (117). Postsymptomatic ALS patients showed some additive improvement in disease progression when treated with 4PBA and taurursodiol (118). Taken together, these studies demonstrate that 4PBA exerts neuroprotective effects in genetically different forms of motor neuron disease *via* an unknown mechanism, although we anticipate that the mechanism is mediated through network interactions. Furthermore, it is unclear at present if the neuroprotective mechanisms are disease-specific or share a common modality.

HDAC6 is highly expressed in the central nervous system and functions by removing acetyl groups from a variety of proteins aside from histones (131). HDAC6 has been implicated in the pathogenesis of many neurodegenerative diseases. Inhibitors of HDAC6 have demonstrated efficacy in ameliorating disease phenotypes in multiple motor neuron diseases. Ablation of *Hdac6* in transgenic mice harboring the ALS-linked *SOD1 (G93A)* mutation improves motor phenotype as well as increases lifespan in these mice (132). Tubastatin A and ACY-738 improve neurite outgrowth and motor neuron function in ALS-linked mutant *FUS* [*FUS (R521H)* and *FUS (P525L)*] motor neurons (119). Tubastatin A improves motor neuron function in ALS-linked *FUS* [*FUS (R521H)* and *FUS (P525L)*] motor neurons cultured in microfluidic devices (120). ACY-738 ameliorates the motor neuron degeneration observed in transgenic mice overexpressing wild-type FUS (127). Tubastatin A improves neurite outgrowth in patient-derived cultured motor neurons harboring different ALS-associated TDP43 mutations [*TARDBP (A382T)*; *TARDBP (G287S)* and *TARDBP (N390S)*] (121). The subcellular distribution of mutant TDP-43 as well as mitochondrial transport along axons were restored by tubastatin A.

In addition to exerting neuroprotective effects in model systems for ALS, HDAC6 inhibitors have been shown to ameliorate motor neuronopathy phenotypes observed in multiple types of axonal peripheral neuropathies, i.e., type 2 CMT. The HDAC6-selective inhibitor SW-100 improves motor phenotype in *MFN2 (R94Q)* mouse model for CMT2A1 (126). Tubastatin A markedly improves motor neuron and muscle innervation in the *HSPB1 (S135F)* mouse model for CMT2F (122). ACY-738 improves survival and ameliorates the motor phenotype observed in CMT2F [*HSPB1 (S135M)*] mice (128, 129). HDAC6 inhibition *via* the novel small molecules T3796106 and T3793168 improves motor neuron phenotype in cultured *HSPB1 (S135M)* CMT motor neurons (130). Tubastatin A improves motor function in the mutant *GARS (R234KY)* as well as in *Gars (C201R)* transgenic mouse models for CMT2D (123). Electrophysiologic misfiring and axonal transport defects are corrected in CMT2D motor neurons by tubastatin A and CKD-504 (124). Tubastatin A and CKD-504 improve deficient neurite outgrowth and defective neuromuscular junction maturation that are observed in zebrafish embryos where *Gars* is knocked down with splice-blocking antisense morpholino oligonucleotides (125).

Combinatory therapeutics have shown promise in animal models for SMA. One of the first examples of a combinatory therapeutic strategy demonstrated additive benefit was treatment of SMA mice with a small-molecule inducer of *SMN2* expression (D156844) alongside a myoprotectant (follistatin) (133). Coadministration of a *SMN2* exon 7 splice-correcting oligonucleotide and RG7800–a small molecule that promotes exon 7 inclusion–provided more pronounced additive effects in SMA mice (134). In addition, combinatory, multi-target therapies using a *SMN2* exon 7 targeted splice correcting antisense oligonucleotide alongside either a broad spectrum histone deacetylase inhibitor (135) or a protein stabilizing agent (136) have shown additive benefits in cell culture and mouse models for SMA. The compounds described previously (4PBA and HDAC6 inhibitors) could potentially be considered as multi-target and multi-modal therapies for MNDs.

## Conclusions and future directions

Lower motor neurons and their connections to skeletal muscles as well as to interneurons–or the motor unit–can be thought of as a network of agents (genes, metabolites and activities) organized into aggregate clusters that interact with each other non-linearly in a hierarchical organization. These agents and their interactions have distinct, adaptable specializations that respond to selective environmental pressure. The complex adaptive system mediates the flow of information or materials from the external input from the lower motor neuron to the neuromuscular junction. Early-onset MNDs can be viewed as consequences of a breakdown of complexity within the motor unit network. A genetic perturbation within an agent in this network would pathologically alter the entire network and the flow of information from the input to the output.

Current strategies for therapeutics development for early-onset MNDs focus on amelioration or replacement of a single perturbation. This reductionist approach has been successful in improving the quality of life and disease presentation in SMA by SMN replacement. While very successful, targeted SMN replacement therapy has provided variable therapeutic benefit within the SMA patient population. Using a complex system approach to understand early-onset MNDs would provide a path for better understanding how loss of a specific agent affects the entire motor system network as well as multiple avenues for therapeutic interventions. These neuroprotective interventions potentially would provide therapeutic benefit for many early-onset MNDs as opposed to a single genetic disorder.

## Data availability statement

The original contributions presented in the study are included in the article/supplementary material, further inquiries can be directed to the corresponding authors.

## Author contributions

All authors listed have made a substantial, direct, and intellectual contribution to the work and approved it for publication.

## Funding

This perspective article has been supported by the Nemours Foundation to MB and RS.

## Conflict of interest

The authors declare that the research was conducted in the absence of any commercial or financial relationships that could be construed as a potential conflict of interest.

## Publisher's note

All claims expressed in this article are solely those of the authors and do not necessarily represent those of their affiliated organizations, or those of the publisher, the editors and the reviewers. Any product that may be evaluated in this article, or claim that may be made by its manufacturer, is not guaranteed or endorsed by the publisher.

## References

[1] Cohen E Bonne G Rivier F Hamroun D. The 2022 version of the gene table of neuromuscular diseases (nuclear genome). Neuromuscul Disord. (2021) 31:1313–57. 10.1016/j.nmd.2021.11.004 34930546

[2] Garg N Park SB Vucic S Yiannikas C Spies J Howells J. Differentiating lower motor neuron syndromes. J Neurol Neurosurg Psychiatry. (2017) 88:474–83. 10.1136/jnnp-2016-313526 28003344 PMC5529975

[3] Shulman LM. Reductionism and the study of neurodegenerative disorders. Mov Disord. (1997) 12:118–9. 10.1002/mds.870120122 8990066

[4] Finkel RS Mercuri E Darras BT Connolly AM Kuntz NL Kirschner J. Nusinersen versus sham control in infantile-onset spinal muscular atrophy. N Engl J Med. (2017) 377:1723–32. 10.1056/NEJMoa1702752 29091570

[5] Mercuri E Darras BT Chiriboga CA Day JW Campbell C Connolly AM. Nusinersen versus sham control in later-onset spinal muscular atrophy. N Engl J Med. (2018) 378:625–35. 10.1056/NEJMoa1710504 29443664

[6] Baranello G Darras BT Day JW Deconinck N Klein A Masson R. Risdiplam in type 1 spinal muscular atrophy. N Engl J Med. (2021) 384:915–23. 10.1056/NEJMoa2009965 33626251

[7] Mendell JR Al-Zaidy S Shell R Arnold WD Rodino-Klapac LR Prior TW. Single-dose gene-replacement therapy for spinal muscular atrophy. N Engl J Med. (2017) 377:1713–22. 10.1056/NEJMoa1706198 29091557

[8] Reilly A Chehade L Kothary R. Curing SMA: are we there yet? Gene Ther. (2022). 10.1038/s41434-022-00349-y. [Epub ahead of print]. 35614235

[9] Martin PB Hicks AN Holbrook SE Cox GA. Overlapping spectrums: the clinicogenetic commonalities between charcot-marie-tooth and other neurodegenerative diseases. Brain Res. (2020) 1727:146532. 10.1016/j.brainres.2019.146532 31678418 PMC6939129

[10] Barabási AL Gulbahce N Loscalzo J. Network medicine: a network-based approach to human disease. Nat Rev Genet. (2011) 12:56–68. 10.1038/nrg2918 21164525 PMC3140052

[11] Sonawane AR Weiss ST Glass K Sharma A. Network medicine in the age of biomedical big data. Front Genet. (2019) 10:294. 10.3389/fgene.2019.00294 31031797 PMC6470635

[12] Manzoni C Lewis PA Ferrari R. Network analysis for complex neurodegenerative diseases. Curr Genet Med Rep. (2020) 8:17–25. 10.1007/s40142-020-00181-z

[13] Sen A Dimlich DN Guruharsha KG Kankel MW Hori K Yokokura T. Genetic circuitry of *Survival motor neuron*, the gene underlying spinal muscular atrophy. Proc Natl Acad Sci U S A. (2013) 110:E2371–80. 10.1073/pnas.1301738110 23757500 PMC3696827

[14] Sanheuza M Chai A Smith C McCray BA Simpson TI Taylor JP. Network analyses reveal novel aspects of ALS pathogenesis. PLoS Genet. (2015) 11:e1005107. 10.1371/journal.pgen.1005107 25826266 PMC4380362

[15] Maeda M Harris AW Kingham BF Lumpkin CJ Opdenaker LM McCahan SM. Transcriptome profiling of spinal muscular atrophy motor neurons derived from mouse embryonic stem cells. PLoS ONE. (2014) 9:e106818. 10.1371/journal.pone.0106818 25191843 PMC4156416

[16] Hensel N Cieri F Santonicola P Tapken I Schüning T Taiana M. Impairment of the neurotrophic signaling hub B-Raf contributes to motoneuron degeneration in spinal muscular atrophy. Proc Natl Acad Sci U S A. (2021) 118:e2007785118. 10.1073/pnas.2007785118 33931501 PMC8106332

[17] Murray LM Beauvais A Gibeault S Courtney NL Kothary R. Transcriptional profiling of differentially vulnerable motor neurons at pre-symptomatic stage in the *Smn*^2*b*/−^ mouse model of spinal muscular atrophy. Acta Neuropathol Commun. (2015) 3:55. 10.1186/s40478-015-0231-1 26374403 PMC4570693

[18] Kline RA Kaifer KA Osman EY Carella F Tiberi A Ross J. Comparison of independent screens on differentially vulnerable motor neurons reveals alpha-synuclein as a common modifier in motor neuron diseases. PLoS Genet. (2017) 13:e1006680. 10.1371/journal.pgen.1006680 28362802 PMC5391970

[19] Brockington A Ning K Heath PR Wood E Kiby J Fusi N. Unravelling the enigma of selective vulnerability in neurodegeneration: motor neurons resistant to degeneration in ALS show distinct gene expression characteristics and decreased susceptibility to excitotoxicity. Acta Neuropathol. (2013) 125:95–109. 10.1007/s00401-012-1058-5 23143228 PMC3535376

[20] Hedlund E Karlsson M Osborn T Ludwig W Isacson O. Global gene expression profiling of somatic motor neuron populations with different vulnerability identify molecules and pathways of degeneration and protection. Brain. (2010) 133:2313–30. 10.1093/brain/awq167 20826431 PMC3139939

[21] Kaplan A Spiller KJ Towne C Kanning KC Choe GT Geber A. Neuronal matrix metalloproteinase-9 is a determinant of selective neurodegeneration. Neuron. (2014) 81:333–48. 10.1016/j.neuron.2013.12.009 24462097 PMC6015650

[22] Nichterwitz S Nijssen J Storvall H Schweingruber C Comley LH Allodi I. LCM-seq reveals unique transcriptional adaptation mechanisms of resistant neurons and identified protective pathways in spinal muscular atrophy. Genome Res. (2020) 30:1083–96. 10.1101/gr.265017.120 32820007 PMC7462070

[23] Langfelder P Horvath S. WGCNA: an R package for weighted correlation network analysis. BMC Bioinformatics. (2009) 9:559. 10.1186/1471-2105-9-559 19114008 PMC2631488

[24] Yang CW Chen CL Chou WC Lin HC Jong YJ Tsai LK. An integrative transcriptomic analysis for identifying novel target genes corresponding to severity spectrum in spinal muscular atrophy. PLoS ONE. (2016) 11:e0157426. 10.1371/journal.pone.0157426 27331400 PMC4917114

[25] Saris CGJ Horvath S van Vught PWJ van Es MA Blauw HM Fuller TF. Weighted gene co-expression network analysis of the peripheral blood from amyotrophic lateral sclerosis patients. BMC Genomics. (2009) 10:405. 10.1186/1471-2164-10-405 19712483 PMC2743717

[26] Li S Zhu Y Wei C Li C Chen W Jiang S. Identification of molecular correlations between DHRS4 and progressive neurodegeneration in amyotrophic lateral sclerosis by gene co-expression network analysis. Front Immunol. (2022) 13:874978. 10.3389/fimmu.2022.874978 35479082 PMC9035787

[27] Chen YZ Bennett CL Huynh HM Blair IP Puls I Irobi J. DNA/RNA helicase gene mutations in a form of juvenile amyotrophic lateral sclerosis (ALS4). Am J Hum Genet. (2004) 74:1128–35. 10.1086/421054 15106121 PMC1182077

[28] Moreira MC Klur S Watanabe M Németh AH Le Ber I Moniz JC. Senataxin, the ortholog of a yeast RNA helicase, is mutant in ataxia-ocular apraxia 2. Nat Genet. (2004) 36:225–7. 10.1038/ng1303 14770181

[29] Fogel BL Cho E Wahnich A Gao F Becherel OJ Wang X. Mutation of senataxin alters disease-specific transcriptional networks in patients with ataxia with oculomotor apraxia type 2. Hum Mol Genet. (2014) 23:4758–69. 10.1093/hmg/ddu190 24760770 PMC4140459

[30] Hadjinicolaou A Ngo KJ Conway DY Provais JP Baker SK Brady LI. De non pathogenic variant in SETX causes a rapidly progessive neurodegenerative disorder of early childhood-onset with severe axonal polyneuropathy. Acta Neuropathol Commun. (2021) 9:194. 10.1186/s40478-021-01277-5 34922620 PMC8684165

[31] Mao Y Kuo SW Chen L Heckman CJ Jiang MC. The essential and downstream common proteins of amyotrophic lateral sclerosis: a protein-protein interaction network analysis. PLoS ONE. (2017) 12:e0172246. 10.1371/journal.pone.0172246 28282387 PMC5345759

[32] Jensen KH Staider AK Wernersson R Roloff-Handschin TC Hansen DH Groenen PMA. A molecular view of amyotrophic lateral sclerosis through the lens of interaction network modules. PLoS ONE. (2022) 17:e0268159. 10.1371/journal.pone.0268159 35576218 PMC9109932

[33] Kumar R Haider S. Protein network analysis to prioritize key genes in amyotrophic lateral sclerosis. IBRO Neurosci Rep. (2022) 12:25–44. 10.1016/j.ibneur.2021.12.002 34918006 PMC8669318

[34] Zhong M Luo Q Ye T Zhu X Chen X Liu J. Identification of candidate genes associated with Charcot-Marie-Toot disease by network and pathway analysis. BioMed Res Int. (2020) 2020:1353516. 10.1155/2020/1353516 33029488 PMC7532371

[35] Bis-Brewer DM Danzi MC Wuchty S Züchner S. A network biology approach to unraveling inherited axonopathies. Sci Rep. (2019) 9:1692. 10.1038/s41598-018-37119-z 30737464 PMC6368620

[36] Van Lent J Verstraelen P Asselbergh B Adriaenssens E Mateiu L Verbist C. Induced pluripotent stem cell-derived motor neurons of CMT type 2 patients reveal progressive mitochondrial dysfunction. Brain. (2021) 144:2471–85. 10.1093/brain/awab226 34128983 PMC8418338

[37] Garcia-Vaquero ML Gama-Carvalho M De Las Rivas J Pinto FR. Searching for overlap between network modules with specific betweeness (S2B) and its application to cross-disease analysis. Sci Rep. (2018) 8:11555. 10.1038/s41598-018-29990-7 30068933 PMC6070533

[38] Gama-Carvalho M Garcia-Vaquero ML Pinto FR Besse F Weis J Voigt A. Linking amyotrophic lateral sclerosis and spinal muscular atrophy through RNA-transcriptome homeostasis: a genomics perspective. J Neurochem. (2017) 141:12–30. 10.1111/jnc.13945 28054357

[39] Chi B O'Connell JD Yamazaki T Gangopadhyay J Gygi SP Reed R. Interactome analysis revealed that the U1 snRNP machinery overlaps with the RNAP II machinery and contains multiple ALS/SMA-causative proteins. Sci Rep. (2018) 8:8755. 10.1038/s41598-018-27136-3 29884807 PMC5993797

[40] Chi B O'Connell JD Iocolano AD Coady JA Yu Y Gangopadhyay J. The neurodegenerative diseases ALS and SMA are linked at the molecular level via the ASC-1 complex. Nucleic Acids Res. (2018) 46:11939–51. 10.1093/nar/gky1093 30398641 PMC6294556

[41] Šoltić D Bowerman M Stock J Shorrock HK Gillingwater TH Fuller HR. Multi-study proteomic and bioinformatic identification of molecular overlap between amyotrophic lateral sclerosis (ALS) and spinal muscular atrophy (SMA). Brain Sci. (2018) 8:212. 10.3390/brainsci8120212 30518112 PMC6315439

[42] Kubinski S Claus P. Protein network analysis reveals a functional connectivity of dysregulated processes in ALS and SMA. Neurosci Insights. (2022) 17:17:26331055221087740. 10.1177/26331055221087740 35372839 PMC8966079

[43] Butchbach MER. Genomic variability in the Survival Motor Neuron genes (*SMN1* and *SMN2*): implications for spinal muscular atrophy phenotype and therapeutics development. Int J Mol Sci. (2021) 22:7896. 10.3390/ijms22157896 34360669 PMC8348669

[44] Oprea GE Kröber S McWhorter ML Rossoll W Müller S Krawczak M. Plastin 3 is a protective modifier of autosomal recessive spinal muscular atrophy. Science. (2008) 320:524–7. 10.1126/science.1155085 18440926 PMC4908855

[45] Stratigopoulos G Lanzano P Deng L Guo J Kaufmann P Darras B. Association of plastin 3 expression with disease severity in spinal muscular atrophy only in postpubertal females. Arch Neurol. (2010) 67:1252–6. 10.1001/archneurol.2010.239 20937953

[46] Yanyan C Yujin Q Jinli B Yuwei J Hong W Fang S. Correlation of PLS3 expression with disease severity in children with spinal muscular atrophy. J Hum Genet. (2014) 59:24–7. 10.1038/jhg.2013.111 24172247

[47] Bernal S Also-Rallo E Martínez-Hernández R Alías L Rodríguez-Alvarez FJ Millán JM. Plastin 3 expression in discordant spinal muscular atrophy (SMA) siblings. Neuromuscul Disord. (2011) 21:413–9. 10.1016/j.nmd.2011.03.009 21546251

[48] Yener IH Topaloglu H Erdem-Özdamar S Dayangac-Erdem D. Transcript levels of *plastin 3* and *neuritin 1* modifer genes in spinal muscular atrophy siblings. Pediatr Int. (2017) 59:53–6. 10.1111/ped.13052 27279027

[49] Wadman RI Jansen MD Curial CAD groen EJN Stam M Wijngaarde CA. Analysis of FUS, PFN2, TDP-43 and PLS3 as potential disease severity modifiers in spinal muscular atrophy. Neurol Genet. (2020) 6:e386. 10.1212/NXG.0000000000000386 32042914 PMC6975178

[50] Ackermann B Kröber S Torres-Benito L Borgmann A Peters M Barkooie SMH. Plastin 3 ameliorates spinal muscular atrophy via delayed axon pruning and improves neuromuscular junction functionality. Hum Mol Genet. (2013) 22:1328–47. 10.1093/hmg/dds540 23263861

[51] McGovern VL Massoni-Laporte A Wang X Le TT Le HT Beattie CE. Plastin 3 expression does not modify spinal muscular atrophy severity in the Δ7 SMA mouse. PLoS ONE. (2015) 10:e0132364. 10.1371/journal.pone.0132364 26134627 PMC4489873

[52] Kaifer KA Villalón E Osman EY Glascock JJ Arnold LL Cornelison DDW. Plastin-3 extends survival and reduces severity in mouse models of spinal muscular atrophy. JCI Insight. (2017) 2:e89970. 10.1172/jci.insight.89970 28289706 PMC5333955

[53] Alrafiah A Karyka E Coldicott I Iremonger K Lewis KE Ning K. Plastin 3 promotes motor axonal growth and extends survival in a mouse model of spinal muscular atrophy. Mol Ther Methods Clin Dev. (2018) 9:81–9. 10.1016/j.omtm.2018.01.007 29552580 PMC5852384

[54] Riessland M Kaczmarek A Schneider S Swoboda KJ Löhr H Bradler C. Neurocalcin Delta suppression protects against spinal muscular atrophy in humans and acress species by restoring impaired endocytosis. Am J Hum Genet. (2017) 100:297–315. 10.1016/j.ajhg.2017.01.005 28132687 PMC5294679

[55] Jiang J Huang J Gu J Cai X Zhao H Lu H. Genomic analysis of a spinal muscular atrophy (SMA) discordant family identifies a novel mutation in *TLL2*, an activator of growth differentiation factor 8 (myostatin): a case report. BMC Med Genet. (2019) 20:204. 10.1186/s12881-019-0935-3 31888525 PMC6938020

[56] Akten B Kye MJ Hao L Wertz MH Singh S Nie D. Interaction of survival of motor neuron (SMN) with HuD proteins with mRNA cpg15 rescues motor neuron axonal deficits. Proc Natl Acad Sci U S A. (2011) 108:10337–42. 10.1073/pnas.1104928108 21652774 PMC3121858

[57] Pla-Martín D Calpena E Lupo V Márquez C Rivas E Sivera R. Junctophilin-1 is a modifier gene of *GDAP1*-related Charcot-Marie-Tooth disease. Hum Mol Genet. (2015) 24:213–29. 10.1093/hmg/ddu440 25168384

[58] He W Bai G Zhou H Wei N White NM Lauer J. CMT2D neuropathy is linked to the neomorphic binding activity of glycyl-tRNA synthetase. Nature. (2015) 526:710–4. 10.1038/nature15510 26503042 PMC4754353

[59] Morelli KH Seburn KL Schroeder DG Spaulding EL Dionne LA Cox GA. Severity of demyelinating and axonal neuropathy mouse models is modified by genes affecting structure and function of peripheral nodes. Cell Rep. (2017) 18:3178–91. 10.1016/j.celrep.2017.03.009 28355569 PMC5415377

[60] Genç B Jara JH Sanchez SS Lagrimas AKB Gözütok Ö Koçak N. Upper motor neurons are a target for gene therapy and UCHL1 is necessary and sufficient to improve cellular integrity of diseased upper motor neurons. Gene Ther. (2022) 29:178–92. 10.1038/s41434-021-00303-4 34853443 PMC9018479

[61] Mentis GZ Blivis D Liu W Drobac E Crowder ME Kong L. Early functional impairment of sensory-motor connectivity in a mouse model of spinal muscular atrophy. Neuron. (2011) 69:453–67. 10.1016/j.neuron.2010.12.032 21315257 PMC3044334

[62] Carlini MJ Triplett MK Pellizzoni L. Neuromuscular denervation and deafferentation but not motor neuron death are disease features in the *Smn*^2*B*/−^ mouse model of SMA. PLoS ONE. (2022) 17:e0267990. 10.1371/journal.pone.0267990 35913953 PMC9342749

[63] Shorrock HK van der Hoorn D Boyd PJ Llavero Hurtado M Lamont DJ Wirth B. UBA1/GARS-dependent pathways drive sensory-motor connectivity defects in spinal muscular atrophy. Brain. (2018) 141:2878–94. 10.1093/brain/awy237 30239612 PMC6158753

[64] Abati E Citterio G Bresolin N Comi GP Corti S. Glial cells involvement in spinal muscular atrophy: could SMA be a neuroinflammatory disease? Neurobiol Dis. (2020) 140:104870. 10.1016/j.nbd.2020.104870 32294521

[65] Kuru S Sakai M Konagaya M Yoshida M Hashizume H Saito K. An autopsy case of spinal muscular atrophy type III (Kugelberg-Welander disease). Neuropathology. (2009) 29:63–7. 10.1111/j.1440-1789.2008.00910.x 18410269

[66] Van den Berg-Vos RM Van den Berg LH Jansen GH Parton M Shaw CE Hesseling-Janssen ALW. Hereditary pure lower motor neuron disease with adult onset and rapid progression. J Neurol. (2001) 248:290–6. 10.1007/s004150170203 11374093

[67] Brockmann K Dreha-Kulaczewski S Dechent P Bönnemann C Helms G Kyllerman M. Cerebral involvement in axonal Charcot-Marie-Tooth neuropathy caused by mitofusin2 mutations. J Neurol. (2008) 255:1049–58. 10.1007/s00415-008-0847-1 18425620

[68] Tarabal O Caraballo-Miralles V Cardona-Rossinyol A Correa FJ Olmos G Lladó J. Mechanisms involved in spinal cord central synapse loss in a mouse model of spinal muscular atrophy. J Neuropathol Exp Neurol. (2014) 73:519–35. 10.1097/NEN.0000000000000074 24806302

[69] Rindt H Feng Z Mazzasette C Glascock JJ Valdivia D Pyles N. Astrocytes influence the severity of spinal muscular atrophy. Hum Mol Genet. (2015) 24:4094–102. 10.1093/hmg/ddv148 25911676 PMC5007659

[70] McGivern JV Patitucci TN Nord JA Barabas MEA Stucky CL Ebert AD. Spinal muscular atrophy astrocytes exhibit abnormal calcium regulation and reduced growth factor production. Glia. (2013) 61:1418–28. 10.1002/glia.22522 23839956 PMC3941074

[71] Ohuchi K Funato M Yoshino Y Ando S Inagaki S Sato A. Notch signaling mediates astrocyte abnormality in spinal muscular atrophy model systems. Sci Rep. (2019) 9:3701. 10.1038/s41598-019-39788-w 30842449 PMC6403369

[72] Martin JE Nguyen TT Grunseich C Nofziger JH Lee PR Fields D. Decreased motor neuron support by SMA astrocytes due to diminished MCP1 secretion. J Neurosci. (2017) 37:5309–18. 10.1523/JNEUROSCI.3472-16.2017 28450545 PMC5456111

[73] Sison SL Patitucci TN Seminary ER Villalon E Lorson CL Ebert AD. Astrocyte-produced miR-146a as a mediator of motor neuron loss in spinal muscular atrophy. Hum Mol Genet. (2017) 26:3409–20. 10.1093/hmg/ddx230 28637335

[74] Zhou C Feng Z Ko CP. Defects in motoneuron-astrocyte interactions in spinal muscular atrophy. J Neurosci. (2016) 36:2543–53. 10.1523/JNEUROSCI.3534-15.2016 26911699 PMC6705489

[75] Ling KKY Lin MY Zingg B Feng Z Ko CP. Synaptic defects in the spinal and neuromuscular circuitry in a mouse model of spinal muscular atrophy. PLoS One. (2010) 5:e15457. 10.1371/journal.pone.0015457 21085654 PMC2978709

[76] Fernandez-Lizarbe S Civera-Tregón A Cantarero L Herrer I Juarez P Hoenicka J. Neuroinflammation in the pathogenesis of axonal Charcot-Marie-Tooth disease caused by lack of GDAP1. Exp Neurol. (2019) 320:113004. 10.1016/j.expneurol.2019.113004 31271761

[77] Khayrullina G Alipio-Gloria ZA Deguise MO Gagnon S Chehade L Stinson M. Survival motor neuron protein deficiency alters microglia reactivity. Glia. (2022) 70:1337–58. 10.1002/glia.24177 35373853 PMC9081169

[78] Vukojicic A Delestrée N Fletcher EV Pagiazitis JG Sankaranarayanan S Yednock TA. The classical complement pathway mediates microglia-dependent remodeling of spinal motor circuits during development and in SMA. Cell Rep. (2019) 29:3087–100. 10.1016/j.celrep.2019.11.013 31801075 PMC6937140

[79] Cerveró C Blasco A Tarabal O Casanovas A Piedrafita L Navarro X. Glial activation and central synapse loss, but not motoneuron degeneration, are prevented by the sigma-1 receptor agonist PRE-084 in the Smn^2B/−^ mouse model of spinal muscular atrophy. J Neuropathol Exp Neurol. (2018) 77:577–97. 10.1093/jnen/nly033 29767748

[80] Mazzocchi F. Complexity and the reduction-holism debate in systems biology. Wiley Interdiscip Rev Syst Biol Med. (2012) 4:413–27. 10.1002/wsbm.1181 22761024

[81] Siegenfeld AF Bar-Yam Y. An introduction to complex systems science and its applications. Complexity. (2020) 2020:6105872. 10.1155/2020/6105872

[82] Turkheimer FE Rosas FE Dipasquale O Martins D Fagerholm ED Expert P. A complex systems perspective on neuroimaging studies of behavior and its disorders. Neuroscientist. (2022) 28:382–99. 10.1177/1073858421994784 33593120 PMC9344570

[83] Scott RC Menendez de la Prida L Mahoney JM Kobow K Sankar R de Curtis M. WONOEP APPRAISAL: the many facets of epilepsy networks. Epilepsia. (2018) 59:1475–83. 10.1111/epi.14503 30009398

[84] Scott RC. Brains, complex systems and therapeutic opportunities in epilepsy. Seizure. (2021) 90:155–9. 10.1016/j.seizure.2021.02.001 33582003 PMC8342615

[85] Blum JA Klemm S Shadrach JL Guttenplan KA Nakayama L Kathiria A. Single-cell transcriptomic analysis of the adult mouse spinal cord reveals molecular diversity of autonomic and skeletal motor neurons. Nat Neurosci. (2021) 24:572–83. 10.1038/s41593-020-00795-0 33589834 PMC8016743

[86] Nedelec S Martinez-Arias A. *In vitro* models of spinal motor circuit's development in mammals: achievements and challenges. Curr Opin Neurobiol. (2021) 66:240–9. 10.1016/j.conb.2020.12.002 33677159

[87] Stifani N. Motor neurons and the generation of spinal motor neuron diversity. Front Cell Neurosci. (2014) 8:293. 10.3389/fncel.2014.00293 25346659 PMC4191298

[88] Enander JMD Jones AM Kirkland M Hurless J Jörntell H Loeb GE. A model for self-organization of sensorimotor function: the spinal monosynaptic loop. J Neurophysiol. (2022) 127:1460–77. 10.1152/jn.00242.2021 35264006 PMC9208450

[89] Enander JMD Loeb GE Jörntell H. A model for self-organization of sensorimotor function: spinal interneuronal integration. J Neurophysiol. (2022) 127:1478–95. 10.1152/jn.00054.2022 35475709 PMC9293245

[90] Pethick J Winter SL Burnley M. Physiological complexity: influence of ageing, disease and neuromuscular fatigue on muscle force and torque fluctuations. Exp Physiol. (2021) 106:2046–59. 10.1113/EP089711 34472160

[91] Laine CM Martinez-Valdes E Falla D Mayer F Farina D. Motor neuron pools of synergistic thigh muscles share most of their synaptic input. J Neurosci. (2015) 35:12207–16. 10.1523/JNEUROSCI.0240-15.2015 26338331 PMC6605305

[92] Del Vecchio A Sylos-Labini F Mondi V Paolillo P Ivanenko Y Lacquaniti F. Spinal motoneurons of the human newborn are highly synchronized during leg movements. Sci Adv. (2020) 6:eabc3916. 10.1126/sciadv.abc3916 33219027 PMC7679172

[93] Hug F Avrillon S Sarcher A Del Vecchio A Farina D. Correlation networks of spinal motor neurons that innervate lower limb muscles during a multi-joint isometric task. J Physiol. (2022). 10.1113/JP283040 35772071

[94] Goldberger AL Peng CK Lipsitz LA. What is physiologic complexity and how does it change with aging and disease? Neurobiol Aging. (2002) 23:23–6. 10.1016/S0197-4580(01)00266-4 11755014

[95] Vaillancourt DE Newell KM. Changing complexity in human behavior and physiology through aging and disease. Neurobiol Aging. (2002) 23:1–11. 10.1016/S0197-4580(01)00247-0 11755010

[96] Finsterer J Aliyev R. Fasciculations in human hereditary disease. Acta Neurol Belg. (2015) 115:91–5. 10.1007/s13760-014-0335-6 25073774

[97] Bashford JA Wickham A Iniesta R Drakakis EM Boutelle MG Mills KR. Accurate interpretation of fasciculation frequency in amyotrophic lateral sclerosis hinges on both muscle type and stage of disease. Brain Commun. (2020) 2:fcaa189. 10.1093/braincomms/fcaa189 33428693 PMC7784041

[98] Bashford JA Wickham A Iniesta R Drakakis EM Boutelle MG Mills KR. The rise and fall of fasciculations in amyotrophic lateral sclerosis. Brain Commun. (2020) 2:fcaa018. 10.1093/braincomms/fcaa018 32901231 PMC7425399

[99] de Carvalho M Kiernan MC Swash M. Fasciculation in amyotrophic lateral sclerosis: origin and pathophysiological relevance. J Neurol Neurosurg Psychiatry. (2017) 88:773–9. 10.1136/jnnp-2017-315574 28490504

[100] Finsterer J Scorza FA. Fasciculation frequency is a questionable biomarker for motor unit loss in amyotrophic lateral sclerosis. Brain Commun. (2020) 2:fcaa188. 10.1093/braincomms/fcaa188 33428690 PMC7784040

[101] Leite MAA Orsini M de Freitas MRG Pereira JS Gobbi FHP Bastos JA. Another perspective on fasciculations: when is it not caused by the classic form of amyotrophic lateral sclerosis or progressive spinal atrophy? Neurol Int. (2014) 6:5208. 10.4081/ni.2014.5208 25309711 PMC4192433

[102] Moosa A Dubowitz V. Spinal muscular atrophy in childhood: two clues to clinical diagnosis. Arch Dis Child. (1973) 48:386. 10.1136/adc.48.5.386 4703068 PMC1648366

[103] Alsaman AS AlShaikh NM. Type III spinal muscular atrophy mimicking muscular dystrophies. Pediatr Neurol. (2013) 48:353–66. 10.1016/j.pediatrneurol.2012.12.027 23583053

[104] dos Santos MAR Brighente SF Massignan A Tenório RB Makariewicz LL Moreira AL. Accuracy of muscle fasciculations for the diagnosis of later-onset spinal muscle atrophy. Neuromuscul Disord. (2022) 32:763–8. 10.1016/j.nmd.2022.07.395 35879189

[105] Rhodes LE Freeman BK Auh S Kokkinis AD La Pean A Chen CH. Clinical features of spinal and bulbar muscular atrophy. Brain. (2009) 132:3242–51. 10.1093/brain/awp258 19846582 PMC2792370

[106] Hensel N Kubinski S Claus P. The need for SMN-independent treatments of spinal muscular atrophy (SMA) to complement SMN-enhancing drugs. Front Neurol. (2020) 11:45. 10.3389/fneur.2020.00045 32117013 PMC7009174

[107] Löscher W. Single-target versus multi-target drugs versus combinations of drugs with multiple targets: preclinical and clinical evidence for the treatment or prevention of epilepsy. Front Pharmacol. (2021) 12:730257. 10.3389/fphar.2021.730257 34776956 PMC8580162

[108] Butchbach MER Lumpkin CJ Harris AW Saieva L Edwards JD Workman E. Protective effects of butyrate-based compounds on a mouse model for spinal muscular atrophy. Exp Neurol. (2016) 279:13–26. 10.1016/j.expneurol.2016.02.009 26892876 PMC4834225

[109] Andreassi C Angelozzi C Tiziano FD Vitali T De Vincenzi E Boninsegna A. Phenylbutyrate increases SMN expression in vitro: relevance for treatment of spinal muscular atrophy. Eur J Hum Genet. (2004) 12:59–65. 10.1038/sj.ejhg.5201102 14560316

[110] Brahe C Vitali T Tiziano FD Angelozzi C Pinto AM Borgo F. Phenylbutyrate increases SMN gene expression in spinal muscular atrophy patients. Eur J Hum Genet. (2005) 13:256–9. 10.1038/sj.ejhg.5201320 15523494

[111] Also-Rallo E Alías L Martínez-Hernández R Caselles L Barceló MJ Baiget M. Treatment of spinal muscular atrophy cells with drugs that upregulate SMN expression reveals inter- and intra-patient variability. Eur J Hum Genet. (2011) 19:1059–65. 10.1038/ejhg.2011.89 21610752 PMC3190259

[112] Mercuri E Bertini E Messina S Pelliccioni M D'Amico A Colitto F. Pilot trial of phenylbutyrate in spinal muscular atrophy. Neuromuscul Disord. (2004) 14:130–5. 10.1016/j.nmd.2003.11.006 14733959

[113] Mercuri E Bertini E Messina S Solari A D'Amico A Angelozzi C. Randomized, double-blind, placebo-controlled trial of phenylbutyrate in spinal muscular atrophy. Neurology. (2007) 68:51–5. 10.1212/01.wnl.0000249142.82285.d6 17082463

[114] Ryu H Smith K Camelo SI Carreras I Lee J Iglesias AH. Sodium phenylbutyrate prolongs survival and regulates expression of anti-apoptotic genes in transgenic amyotrophic lateral sclerosis mice. J Neurochem. (2005) 93:1087–98. 10.1111/j.1471-4159.2005.03077.x 15934930

[115] Petri S Kiaei M Kipiani K Chen J Calingasan NY Crow JP. Additive neuroprotective effects of a histone deacetylase inhibitor and a catalytic antioxidant in a transgenic mouse model of amyotrophic lateral sclerosis. Neurobiol Dis. (2006) 22:40–9. 10.1016/j.nbd.2005.09.013 16289867

[116] Del Signore SJ Amante DJ Kim J Stack EC Goodrich S Cormier K. Combined riluzole and sodium phenylbutyrate therapy in transgenic amyotrophic lateral sclerosis mice. Amyotroph Lateral Scler. (2009) 10:85–94. 10.1080/17482960802226148 18618304

[117] Cudkowicz ME Andres PL MacDonald SA Bedlack R Coudry R Brown RH Jr. Phase 2 study of sodium phenylbutyrate in ALS. Amyotroph Lateral Scler. (2009) 10:99–106. 10.1080/17482960802320487 18688762

[118] Paganoni S Macklin EA Hendrix S Berry JD Elliott MA Maiser S. Trial of sodium phenylbutyrate-taurursodiol for amyotrophic lateral sclerosis. N Engl J Med. (2020) 383:919–30. 10.1056/NEJMoa1916945 32877582 PMC9134321

[119] Guo W Naujock M Fumagalli L Vandoorne T Baatsen P Boon R. HDAC6 inhibition reverses axonal transport defects in motor neurons derived from FUS-ALS patients. Nat Commun. (2017) 8:861. 10.1038/s41467-017-00911-y 29021520 PMC5636840

[120] Stoklund Dittlau K Krasnow EN Fumagalli L Vandoorne T Baatsen P Kerstens A. Human motor units in microfluidic devises are impaired by *FUS* mutations and improved by HDAC6 inhibition. Stem Cell Rep. (2021) 16:2213–27. 10.1016/j.stemcr.2021.03.029 33891869 PMC8452598

[121] Fazal R Boeynaems S Swijsen A De Decker M Fumagalli L Moisse M. HDAC6 inhibition restores TDP-43 pathology and axonal transport defects in human motor neurons with *TARDBP* mutations. EMBO J. (2021) 40:e106177. 10.15252/embj.2020106177 33694180 PMC8013789

[122] d'Ydewalle C Krishnan J Chiheb DM Van Damme P Irobi J Kozikowski AP. HDAC6 inhibitors reverse axonal loss in a mouse model of mutant HSPB1-induced Charcot-Marie-Tooth disease. Nat Med. (2011) 17:968–74. 10.1038/nm.2396 21785432

[123] Mo Z Zhao X Liu H Chen XQ Pham J Wei N. Aberrant GlyRS-HDAC6 interaction linked to axonal transport deficits in Charcot-Marie-Tooth neuropathy. Nat Commun. (2018) 9:1007. 10.1038/s41467-018-03461-z 29520015 PMC5843656

[124] Smith AST Kim JH Chun C Gharai A Moon HW Kim EY. HDAC6 inhibition corrects electrophysiological and axonal transport deficits in a human stem cell-based model of Charcot-Marie-Tooth disease (type 2D). Adv Biol. (2022) 6:2101308. 10.1002/adbi.202101308 34958183 PMC8849597

[125] Jeong HS Kim HJ Kim DH Chung KW Choi BO Lee JE. Therapeutic potential of CKD-504, a novel selective histone deacetylase 6 inhibitor, in a zebrafish model of neuromuscular junction disorders. Mol Cells. (2022) 45:231–42. 10.14348/molcells.2022.5005 35356895 PMC9001154

[126] Picci C Wong VSC Costa CJ McKinnon MC Goldberg DC Swift M. HDAC6 inhibition promotes α-tubulin acetylation and ameliorates CMT2A peripheral neuropathy in mice. Exp Neurol. (2020) 328:113281. 10.1016/j.expneurol.2020.113281 32147437

[127] Rossaert E Pollari E Jaspers T Van Helleputte L Jarpe M Van Damme P. Restoration of histone acetylation ameliorates disease and metabolic abnormalities in a FUS mouse model. Acta Neuropathol Commun. (2019) 7:107. 10.1186/s40478-019-0750-2 31277703 PMC6612190

[128] Shen S Benoy V Bergman JA Kalin JH Frojuello M Vistoli G. Bicyclic-capped histone deacetylase 6 inhibitors with improved activity in a model of axonal Charcot-Marie-Tooth disease. ACS Chem Neurosci. (2016) 7:240–58. 10.1021/acschemneuro.5b00286 26599234 PMC4775085

[129] Benoy V Vanden Berghe P Jarpe M Van Damme P Robberecht W Van Den Bosch L. Development of improved HDAC6 inhibitors as pharmacological therapy for axonal Charcot-Marie-Tooth disease. Neurotherapeutics. (2017) 14:417–28. 10.1007/s13311-016-0501-z 27957719 PMC5398982

[130] Adalbert R Kaieda A Antoniou C Loreto A Yang X Gilley J. Novel HDAC6 inhibitors increase tubulin acetylation and rescue axonal transport of mitochondria in a model of Charcot-Marie-Tooth type 2F. ACS Chem Neurosci. (2020) 11:258–67. 10.1021/acschemneuro.9b00338 31845794 PMC7614726

[131] LoPresti P. HDAC6 in diseases of cognition and of neurons. Cells. (2021) 10:12. 10.3390/cells10010012 33374719 PMC7822434

[132] Taes I Timmers M Hersmus N Bento-Abreu A Van Den Bosch L Van Damme P. *Hdac6* deletion delays disease progression in the *SOD1*^*G*93*A*^ mouse model of ALS. Hum Mol Genet. (2013) 22:1783–90. 10.1093/hmg/ddt028 23364049

[133] Harris AW Butchbach MER. The effect of the DcpS inhibitor D156844 on the protective action of follistatin in mice with spinal muscular atrophy. Neuromuscul Disord. (2015) 25:699–705. 10.1016/j.nmd.2015.05.008 26055638 PMC4540686

[134] Kray KM McGovern VL Chugh D Arnold WD Burghes AHM. Dual SMN inducing therapies can rescue survival and motor unit function in symptomatic Δ7 SMA mice. Neurobiol Dis. (2021) 159:105488. 10.1016/j.nbd.2021.105488 34425216 PMC8502210

[135] Marasco LE Dujardin G Sousa-Luís R Liu YH Stigliano JN Nomakuchi T. Counteracting chromatin effects of a splicing-correcting antisense oligonucleotide improves its therapeutic efficacy in spinal muscular atrophy. Cell. (2022) 185:2057–70. 10.1016/j.cell.2022.04.031 35688133 PMC9555286

[136] Dumas SA Villalón E Bergman EM Wilson KJ Marugan JJ Lorson CL. A combinatorial approach increases SMN level in SMA model mice. Hum Mol Genet. (2022) 31:2984–3000. 10.1093/hmg/ddac068 35419606 PMC9433732

